# Implementation of Speed-Efficient Key-Scheduling Process of AES for Secure Storage and Transmission of Data

**DOI:** 10.3390/s21248347

**Published:** 2021-12-14

**Authors:** Thanikodi Manoj Kumar, Kavitha Rani Balmuri, Adam Marchewka, Parameshachari Bidare Divakarachari, Srinivas Konda

**Affiliations:** 1 Department of Electronics and Communication Engineering, Karpagam Institute of Technology, Coimbatore 641105, Tamil Nadu, India; manojkumar.pla@karpagamtech.ac.in; 2 Department of Information Technology, CMR Technical Campus, Hyderabad 501401, Telangana, India; kavitharani.cse@cmrtc.ac.in; 3 Faculty of Telecommunications, Computer Science and Electrical Engineering, Bydgoszcz University of Science and Technology, 85-796 Bydgoszcz, Poland; 4 Department of Telecommunication Engineering, GSSS Institute of Engineering and Technology for Women, Mysuru 570016, India; paramesh@gsss.edu.in; 5 Department of Computer Science Engineering, CMR Technical Campus, Kandlakoya, Hyderabad 501401, India; Srinivas.cse@cmrtc.ac.in

**Keywords:** advanced encryption standard, data security, field programmable gate arrays, fork–join model of key expansion, hardware resources, propagation delay

## Abstract

Nowadays, a large number of digital data are transmitted worldwide using wireless communications. Therefore, data security is a significant task in communication to prevent cybercrimes and avoid information loss. The Advanced Encryption Standard (AES) is a highly efficient secure mechanism that outperforms other symmetric key cryptographic algorithms using message secrecy. However, AES is efficient in terms of software and hardware implementation, and numerous modifications are done in the conventional AES architecture to improve the performance. This research article proposes a significant modification to the AES architecture’s key expansion section to increase the speed of producing subkeys. The fork–join model of key expansion (FJMKE) architecture is developed to improve the speed of the subkey generation process, whereas the hardware resources of AES are minimized by avoiding the frequent computation of secret keys. The AES-FJMKE architecture generates all of the required subkeys in less than half the time required by the conventional architecture. The proposed AES-FJMKE architecture is designed and simulated using the Xilinx ISE 5.1 software. The Field Programmable Gate Arrays (FPGAs) behaviour of the AES-FJMKE architecture is analysed by means of performance count for hardware resources, delay, and operating frequency. The existing AES architectures such as typical AES, AES-PNSG, AES-AT, AES-BE, ISAES, AES-RS, and AES-MPPRM are used to evaluate the efficiency of AES-FJMKE. The AES-FJMKE implemented using Spartan 6 FPGA used fewer slices (i.e., 76) than the AES-RS.

## 1. Introduction

Nowadays, the growth of lightweight, robust, and effective encryption algorithms are required to provide network security for information technology applications. The developed encryption algorithms are essential for maximizing the throughput and data size of IoT, and it is used in mobile transmissions, video streaming, real-time communications, and so on [1,2,3,4,5,6]. The methods of encryption/decryption are classified into two types such as symmetric and asymmetric methods. In that, symmetric cryptography uses only one key for encryption and decryption, whereas asymmetric cryptography uses two distinct keys to accomplish the encryption and decryption [7,8,9]. Symmetric cryptography is extensively used among all cryptographic methods due to its low energy necessities and simplicity. Hence, the symmetric block cipher, namely AES, was developed in 2001 by the National Institute of Standards and Technology (NIST); however, this AES is an alteration for the typical data encryption standard [10,11,12,13].

Some of block cipher algorithms used in the communication applications are Khudra [14], KASUMI [15], LRBC [16], PRESENT [17], SLIM [18], HIGHT [19], Simon [20], KLEIN [21], Midori [22], and CLEFIA [23]. However, the lightweight cipher affects the performance when the system has a huge number of devices during the communication [24]. Additionally, the lightweight block ciphers offer only a lower level of security than the conventional algorithms [25]. Authentication, confidentiality, and integrity are considered as the significant objectives of the cryptographic protocols. The AES is served as a significant cryptographic algorithm, whereas it satisfies the essential security goals of availability, confidentiality, and integrity during the communication on the insecure transmission medium [26,27]. Since the cryptographic process with an extreme computation complexity of ciphers avoids the key from the attempt of brute force [28]. The AES is implemented in different hardware platforms such as graphics processing units, embedded processors, ASIC, and FPGA because of its extensive utilization [29]. The configuration of hardware units using FPGA’s reconfigurable logic resources is used for achieving high pipelining and parallelism. The balancing over the pipeline is obtained by adding and relocating the registers. The usage of multiple ports in the memory units is used to increase the speed of the read/write operations [30].

FPGA chips can operate simultaneously and it has a comparatively flexible architecture. Hence, the test cycles and design cost of the FPGA chips are lower [31,32,33,34]. Since two different LUT-based methods, namely substitution box (S-Box) and T-box, are used for an effective design of AES over the FPGA, LUT-based encryption and decryption are not only memory intensive but also asymmetric because of its transformation sequence and AES functions of encryption and decryption. Hence, the process of encryption and decryption are designed individually, and it occupies a significant amount of BRAM over the LUT-based AES [35]. However, the clock speed, library, and throughout are difficult to achieve by AES design due to its complexity, its user scheduling process, and the dynamic nature of its design. Since the S-box of the AES’s sub-byte process consumes more power than the other modules of the circuit [36]. The multiplicative inversion used in the sub-byte transformation requires higher resources and finite field arithmetic [37].

Some of the conventional AES architectures are described as below:

Benhadjyoussef et al. [38] presented the fault-resistant method for securing the AES against attacks. Parity checking was used to develop the error detection for the time redundancy of subbytes function and linear operations. Specifically, the error detection code depends on the cyclic redundancy check that was used to identify the parity of the Shift Rows, Mix Columns, and Add Round Key functions. On the other hand, faults were inserted in the SubBytes transformation to identify the temporal redundancy. However, the information redundancy method caused high overhead, which affects the system performance. Sheikhpour, Ko, and Mahani, [39] developed the 32-bit AES encryption/decryption for IoT and resource-constrained applications. Here, the low-cost fault-resilient structure was developed for the data path. Subsequently, an on-the-fly key expansion unit was also designed for the key generation of encryption/decryption processes. Here, the area was minimized using resource-sharing among encryption and decryption operations. Sikka et al. [40] presented the design of the AES for automotive applications. In this work, the 128-bit key of AES was designed using the High-Level Synthesis (HLS) tool. Specifically, HLS was based on the bit widths while designing the AES over the FPGA. However, the re-computation of the signal width increased the overall latency of the AES algorithm. 

Zodpe and Sapkal [41] presented the PN Sequence Generator (PNSG) for creating the S-box and initial keys for Encryption/Decryption. Here, the Linear Feedback Shift Register (LFSR) was used for designing the PNSG, whereas the LFSR was represented using the generator polynomial. The designed PNSG was used to offer different random number sequences by using the initial seed and feedback tap. The robustness of the AES cryptography was enhanced by using the feature of PNSG. However, the design of AES using non-pipelined stages required high hardware resources. Shahbazi and Ko [42] presented the 128-bit of AES in counter mode for high traffic applications. Inner and outer pipelining methods were used to achieve high throughput, and an affine transformation (AT) method was designed to minimize the area. The developed affine transformation was the hybrid method of affine and inverse isomorphic transformation. In AES, the operations of Sub-Bytes and Shift Rows were swapped, and then Add Round Key was combined with the Shift Rows. Moreover, the Mix Column operation was divided into two distinct phases for achieving the latency. However, the accomplishment of mix column in one clock caused high latency. Madhavapandian and MaruthuPandi [43] developed q 128-bit AES cryptographic method for securing the Transmission Control Protocol/Internet Protocol (TCP/IP). An effective mix column Boolean Expression (BE) using gate replacement and resource sharing structure was used to modify the mix column operation. Accordingly, the optimized architecture of AES was used to minimize power consumption. However, it was required that the time complexity be minimized because it increased the delay during the communication using TCP/IP protocol. 

Arul et al. [44] developed the Iterative Structure of the AES (ISAES) for lessening the hardware resources. The architecture of renovated S-box was used in the AES to minimize the area. Here, the usage of LUT in the composite field arithmetic was accomplished in the multiplication operation. Moreover, the Vedic multiplier was employed in the Mix Column transformation, which was used to decrease the hardware resources of AES. However, the operating frequency of the AES was less because of the high delay. Wegener et al. [45] developed AES S-box by using the function of the Rotational Symmetry (RS). In this work, AES was designed by using the internal MUXes and slice registers, and this AES does not require any Block RAM (BRAM). The Boolean masking with a less amount of two shares over an AES’s decomposition was applied to generate the higher robustness against the attacks. Here, the masked AES design was used to optimize the LUT implementation. However, the replication of linear operators and their independent operation was increased the overall area of AES. Kumar [46] developed the architecture of MPPRM for designing the AES’s SubBytes/InvSubBytes transformation. These transformations were utilized for designing the subpipelining architecture. The hardware resources such as AND and XOR gates were reduced using the MPPRM in SubBytes and InvSubBytes transformations. Here, a 128-bit key was generated by the key expansion structure, and this key was given to the subpipelined structure. Due to the utilization of the delay module in the output of AND gate, a high-speed encryption/decryption was achieved in AES. Here, the AES’s area was increased because of the recurrent key generation in the encryption process. 

The problems of the conventional AES are stated as follows: The frequent computation of input, output, and intermediate signal width leads to an increase in the delay of the AES [40]. The hardware resources also increased because of AES design using non-pipelined stages [41]. Due to the high delay, the operating frequency is decreased in the AES architecture [44]. The area of the overall AES is increased because of the replication of linear function and independent operation [45]. Hence, the AES is developed with an effective FJMKE architecture to avoid the aforesaid issues. The FJMKE architecture is used to create the multiple subkeys simultaneously, which helps to decrease the delay in the AES. The generation of multiple subkeys using the FJMKE leads to reducing the combinational logics as it avoids frequent calculation subkeys. 

The conventional AES architecture generates the subkeys according to the previous step subkeys, whereas the proposed AES-FJMKE architecture generates the subkeys only based on the main key that helps to reduce the overall propagation delay.

The research contributions are as follows:

- In this research, the FJMKE architecture is used to generate the subkeys in a parallel way, whereas the generation of the subkeys does not depend on the subkeys from the previous step this leads to minimize the propagation delay. This multiple subkey generation decreases the delay while encrypting the plain text.
- The combinational logic of the overall AES is minimized by avoiding the frequent computation of secret keys using FJMKE architecture, which lessens AES’s resources.
- There are six different FPGA devices, namely Virtex 4, Virtex 5, Virtex 6, Spartan 3, Spartan 6, and Kintex 7, that are used for analysing the AES-FJMKE architecture.

The organization of this research article is as follows. Section 2 explains the typical AES architecture. Section 3 describes the AES-FJMKE architecture in detail. The performance evaluation of the AES-FJMKE architecture is discussed in Section 4. Further, Section 5 discusses the research’s conclusion and future endeavours.

## 2. Related Works

This section provides information about the conventional AES algorithm along with its encryption and decryption processes.

### 2.1. Advanced Encryption Standard

In the AES cryptographic algorithm [47,48,49,50,51], only one secret key is employed for encrypting and decrypting plain texts. AES can be classified as AES-128, AES-192, or AES-256, depending on the key sizes used in encryption and decryption operations, whereas the number denotes the number of bits that exist in the secret key in the AES versions. All versions of the AES process input plain text in a step-by-step fashion. The number of rounds for AES varies between versions and is dependent on the key size, as shown in Table 1.

Separate subkeys are used for each round of AES operation. Indeed, these subkeys are generated from the primary original key via a process called key scheduling. In all three AES variants, the processing of input data is 128 bits. The term “number of rounds” refers to the number of times a single data block is encrypted and decrypted using different subkeys (one at a time) obtained during the key expansion process. All operations are identical throughout the operation’s rounds. Before beginning the encryption operation, a pre-round transformation is performed using the primary original secret key, and the other subkeys are utilized in each round.

### 2.2. Encryption and Decryption of AES

AES uses a secret key to encrypt a 128-bit of plain text to generate the ciphertext. The AES performs all four operations in all rounds except the last round. The final round of encryption and the initial round of decryption will not use Mix Columns. A plain text string of 128 bits in length is ordered as a 4 × 4 state matrix, with each element represented by a byte.

- Substitution;
- Shift Rows;
- Mix columns;
- Add round key.

#### 2.2.1. Substitution

A byte value is substituted for other bytes in this process. The AES algorithm contains only one non-linear process: substitution. The core processes of substitution are matrix multiplication and affine transformation. By replacing the Rijndael S-box byte value directly, the decryption process employs inverse S-box substitution.

#### 2.2.2. Shift Rows

The second, third, and fourth rows of the state matrix are left shifted 1, 2, and 3 times, respectively. The first row of the state matrix remains unchanged. In addition, the right shift operation is carried out on the rows during the decryption process.

#### 2.2.3. Mix Columns 

In this phase, the alteration is performed in the column. The simple function is matrix multiplication. Equations (1) and (2) shows the function of Mix column and Inv Mix column.

(1) $$\left[\begin{array}{c} S’1\\ S’2\\ S’3\\ S’4 \end{array}\right]=\left[\begin{array}{c} 02\;\;\;\;\;\;\;\;03\;\;\;\;\;\;\;\;\;\;01\;\;\;\;\;\;\;\;\;\;01\\ 01\;\;\;\;\;\;\;\;02\;\;\;\;\;\;\;\;\;\;03\;\;\;\;\;\;\;\;\;\;01\\ 01\;\;\;\;\;\;\;\;01\;\;\;\;\;\;\;\;\;\;02\;\;\;\;\;\;\;\;\;\;03\\ 03\;\;\;\;\;\;\;\;01\;\;\;\;\;\;\;\;\;\;01\;\;\;\;\;\;\;\;\;\;02 \end{array}\right]\left[\begin{array}{c} S1\\ S2\\ S3\\ S4 \end{array}\right]$$

(2) $$\left[\begin{array}{c} S’1\\ S’2\\ S’3\\ S’4 \end{array}\right]=\left[\begin{array}{c} 0e\;\;\;\;\;\;\;\;0b\;\;\;\;\;\;\;\;\;\;0d\;\;\;\;\;\;\;\;\;\;09\\ 09\;\;\;\;\;\;\;\;0e\;\;\;\;\;\;\;\;\;\;0b\;\;\;\;\;\;\;\;\;\;0d\\ 0d\;\;\;\;\;\;\;\;09\;\;\;\;\;\;\;\;\;\;0e\;\;\;\;\;\;\;\;\;\;0b\\ 0b\;\;\;\;\;\;\;\;0d\;\;\;\;\;\;\;\;\;\;09\;\;\;\;\;\;\;\;\;\;0e \end{array}\right]\left[\begin{array}{c} S1\\ S2\\ S3\\ S4 \end{array}\right]$$

where S’1, S’2, S’3, S’4 are the output obtained after mix column operation and S1, S2, S3, S4 are the input given to the mix column process.

#### 2.2.4. Add Round Key 

The secret key performs its actual function during the Add Round Key step. All the preceding operations are easily reversible. Before initiating the Add Round Key process, the secret and all subkeys generated during the key expansion process are organized as a 4 × 4 state matrix. The Add Round Key step’s core process is the modulo EXOR addition between the key’s state matrix and the output of the mix column operation. The ciphertext is formed by the output of the added round key of the final round of encryption. These four operations are repeated according to the number of rounds of operation specified for the various AES versions. A separate subkey must be used for each round of operation, and the subkey information must be kept confidential. The main secret key is identified easily when the unauthorized person knows about the subkey’s information. This research focuses primarily on the critical scheduling process.

### 2.3. Existing Key Expansion Architecture 

**Figure 1:**
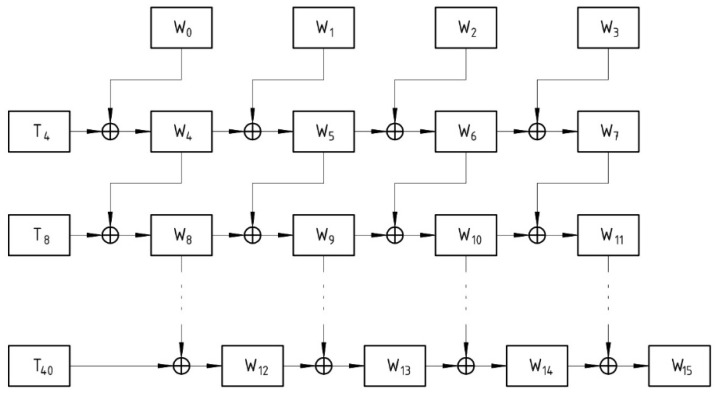
Typical key expansion architecture.

AES-128 is considered in this research, and the detailed key scheduling process for conventional AES-128 is explained in detail. The critical scheduling process for AES-128 is depicted in Figure 1. All operations in the AES key expansion process are performed at the word level. Thus, the 128-bit primary secret key is divided into four 32-bit words. As shown in Table 1, the encryption operation for AES-128 requires ten subkeys. The most intriguing aspect of this key expansion architecture is the interdependence of the subkeys, which prevents subkey predictability. 

Due to the fact that mathematical operations are performed on words, a subkey can be created by combining four consecutive words. For instance, in Figure 1, W4, W5, W6, and W7 are combined to create the first subkey. AES 128 generates 44 words as a result of the key scheduling process. The first four words are derived from the main one and are used in the round prior to transformation. The remaining 40 words are divided into 10 distinct subkeys. T4 denotes a temporary word. T8 denotes a permanent word. The key expansion architecture is nonlinear due to the generation process of temporary words. 

The operations for creating temporary words include S-Box substitution, word rotation, and the EXOR operation with a constant value. The following equation explains how temporary words are created, and Table 2 lists the R constant values (Rcon) for each round. *Temporary words* are formed from the previous subkey’s final word, as shown in Equation (3).

(3) Temporary word=Subword (Rotword(Wi−1))⊕ Rcon

where Rotword is used to perform onebyte circulr shift on the word (Wi).

## 3. AES-FJMKE Architecture

**Figure 2:**
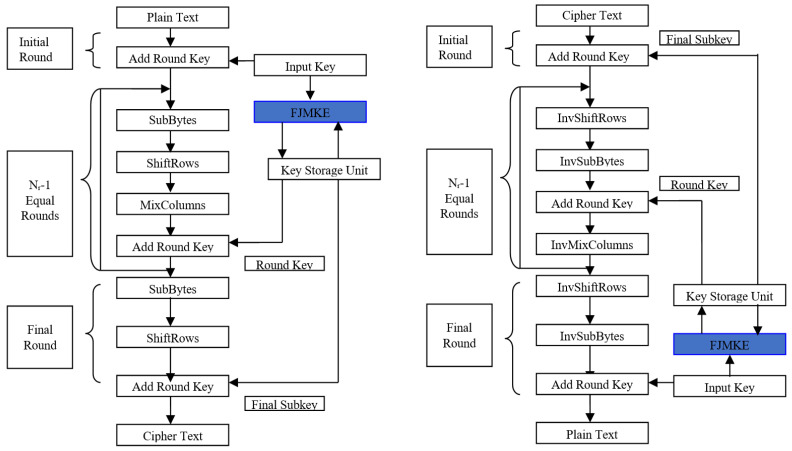
Architecture of AES-FJMKE.

In the proposed AES-FJMKE architecture, an effective key expansion design is developed using the fork-join model. The developed FJKME is used to generate multiple keys at a time. These multiple keys are used to encrypt different sets of plain text that help to minimize the delay. Accordingly, the multiple key generations using FJKME lead to minimizing the combinational blocks of overall AES architecture. Moreover, the other operations, namely Substitution, Shift Rows, Mix Columns, and Add Round Key are similar to the conventional AES architecture. The main objective of this AES-FJMKE architecture is to achieve less propagation delay while achieving security. The overall architecture of AES using FJKME is illustrated in Figure 2. 

### FJMKE Architecture 

**Figure 3:**
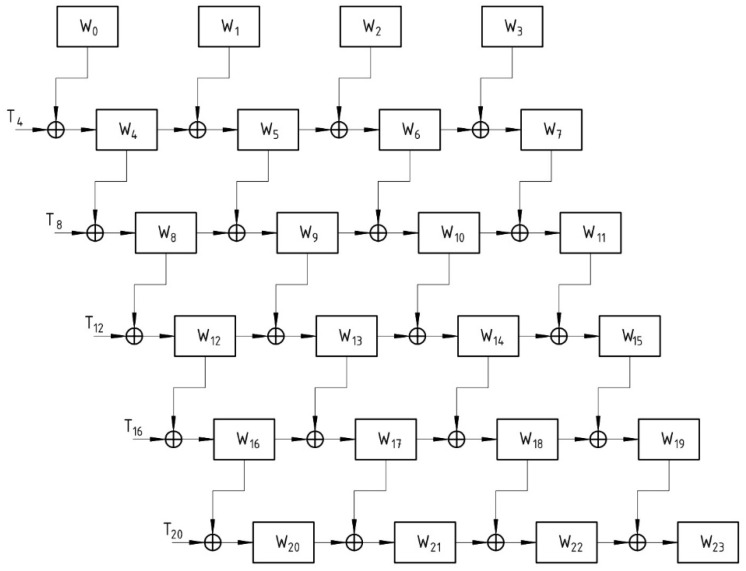
FJMKE for AES-128.

The nominal AES employs a sequential key-scheduling process that generates all subkeys sequentially. The concurrent generation of subkeys is not possible due to the dependency of the temporary word for each subkey over the final word of the preceding subkey. However, interdependence between subkeys is critical in maintaining the secrecy of subkeys. In a traditional architecture, the final word is generated after all previous words have been generated, which is a time-consuming process. With the modifications made to the key scheduling process, the AES-FJMKE architecture aims to reduce time delays. Sequential processes can be made simultaneous by incorporating additional circuitry into the conventional architecture. All subkeys are concurrently generated in this AES architecture, and the current subkey does not require waiting for the previous subkey generation. The time required to generate the subkeys is minimized by using the FJMKE. The structure of FJMKE within the block remains sequential; therefore, the subkeys are sequentially generated in AES. The time consumption for generating the subkeys in AES is high when the overall key expansion is performed at one time. Therefore, the entire architecture of AES is split into two parallel blocks in this fork–join model to reduce the time required to generate subkeys. For AES-128, the first block generates the first five subkeys, while the second block generates the remaining five subkeys. This architecture differs slightly from conventional architecture in that the sixth subkey is dependent on the main key rather than the fifth subkey. In conventional AES architecture, the dependency between the subkeys is high, while generating the subkeys for successive rounds increases the delay. However, the designed FJMKE architecture only depends on the main key during the subkey generation, which lessens the propagation delay for the AES. When compared to the conventional architecture, this modification reduces the total execution time for generating ten subkeys by half. After completing the subkey generation, the subkeys from these two blocks are concatenated together as ten subkeys. From these ten subkeys (i.e., ten different outputs), each subkey is taken for each round to accomplish the encryption/decryption processes. Figure 3 shows the designed key expansion architecture of AES.

**Figure 4:**
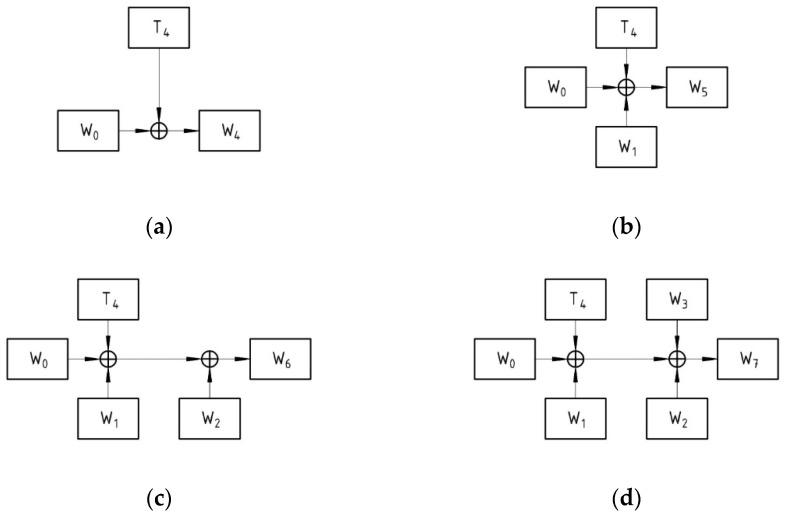
Subkeys generation, (**a**) W4, (**b**) W5, (**c**) W6, and (**d**) W7.

For example, the generation of W4, W5, W6, and W7 in the FJMKE is illustrated in Figure 4. In conventional AES architecture, the generation of W6 depends on the W5 computed from the previous step. Therefore, the generation of current subkeys has to wait until the completion of previous step subkey generation. Hence, the subkey generation of the conventional AES requires more clock cycles. On the other hand, the proposed FJMKE architecture generates all subkeys in a parallel way without waiting for any subkey from the previous step, which minimizes the propagation delay. Specifically, the generation of W6 does not require waiting until the completion of W5 generation. On the contrary, the designed FJMKE architecture generates the subkeys by using main secret keys, which lessens the propagation delay. For example, the conventional AES requires four clock cycles for generating the subkeys W4, W5, W6, and W7, while the FJMKE requires only one clock cycle for generating the subkeys W4, W5, W6, and W7 as it performs concurrent subkey generation. The logical elements used in the AES-FJMKE architecture is slightly higher than the conventional AES which is within an acceptable level. However, this slight increment in logics does not create an impact on the overall AES-FJMKE architecture because the designed FJMKE based key expansion is used only one time during the encryption/decryption processes. Therefore, the interdependence between subkeys is avoided and the propagation delay for producing subkeys is significantly minimized with the help of a circuit built on the basis of the following Equations (4)–(7).

(4) $$W_{4}=T_{4}⊕W_{0}$$

(5) $$W_{5}=T_{4}⊕W_{0}⊕W_{1}$$

(6) $$W_{6}=T_{4}⊕W_{0}⊕W_{1}⊕W_{2}$$

(7) $$W_{7}=T_{4}⊕W_{0}⊕W_{1}⊕W_{2}⊕W_{3}$$

Similarly, multiple keys are generated from the designed FJMKE architecture, and it is used to accomplish the encryption over multiple plain texts. For example, the generation of W15 using the FJMKE architecture is expressed in the following Equation (8).

(8) $$W_{15}=T_{12}⊕T_{8}⊕T_{4}⊕W_{0}⊕T_{8}⊕T_{4}⊕W_{0}⊕T_{4}⊕W_{0}\;⊕W_{1}⊕T_{8}⊕T_{4}⊕W_{0}⊕T_{4}⊕W_{0}⊕W_{1}⊕T_{4}\;⊕W_{0}⊕W_{1}⊕W_{2}⊕T_{8}⊕T_{4}⊕W_{0}⊕T_{4}⊕W_{0}⊕W_{1}\;⊕T_{4}⊕W_{0}⊕W_{1}⊕W_{2}⊕T_{4}⊕W_{0}⊕W_{1}⊕W_{2}⊕W_{3}$$

The concept underlying the preceding expression is to perform all necessary mathematical operations at each and every step. Rather than using the result from the previous step, the result is obtained by recalculating. While this process requires more space and energy, the total time required to produce the end result is reduced. In a conventional architecture, each step or process of subkey generation must be delayed until the previous subkey generation process is complete. However, in this AES-FJMKE architecture, there is no requirement to wait until the previous word of the subkey is complete before beginning the generation of the current word of the subkey. This significantly reduces the propagation delay associated with generating the required number of subkeys. The model described above can be extended to include all ten subkey generation processes.

The architecture of the modified subkey generation process using the FJMKE is illustrated in Figure 4. All four words in the first subkey, W4, W5, W6, and W7, can be generated concurrently. There is no reason to delay the process of generating words W5 until the block that generates W4 is executed. Additionally, processes for generating W6 and W7 can be started concurrently with the process for generating words W4. This property qualifies this architecture for applications that require rapid execution. The AES-FJMKE architecture retains all of the conventional algorithm’s diffusion and confusion operations. 

## 4. Results and Discussion

The results of the proposed AES-FJMKE are described in this section. The synthesis and simulation of the AES-FJMKE architecture is done using the Xilinx ISE 5.1 software. Here, the Hardware Description Language (HDL) is utilized for designing the AES architecture. For this AES architecture, the FJMKE architecture is developed for lessening the delay while decreasing the hardware resources. Moreover, the developed AES-FJMKE architecture is employed for processing the 128-bit of plaintext.

### 4.1. Performance Evaluation for AES-FJMKE 

The design and evaluation of AES-FJMKE is made by using six distinct FPGA devices, namely Virtex 4, Virtex 5, Virtex 6, Spartan 3, Spartan 6, and Kintex 7. The performance of the AES-FJMKE is evaluated by means of performance count for hardware resources, delay, and operating frequency. The evaluation of the results of the AES for different FPGA devices is shown in the tables below.

**Figure 5:**
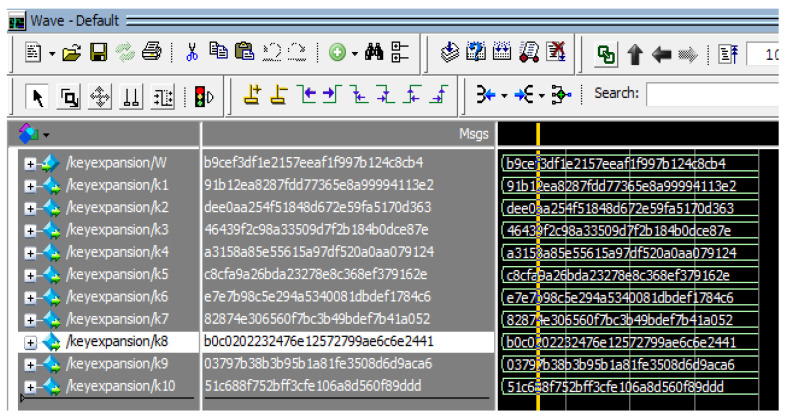
Simulation waveform of generated keys of AES-FJMKE designed in Virtex 4.

**Figure 6:**
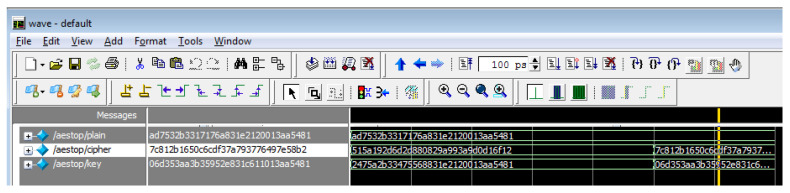
Overall simulation waveform of AES-FJMKE architecture.

The hardware utilization analysis of the AES-FJMKE developed in the Virtex, Spartan, and Kintex devices are shown in Table 3, Table 4 and Table 5, respectively. Moreover, the delay and operating frequency evaluation for the AES-FJMKE is shown in Table 6, where the delay and operating frequency are the time consumption-related-parameters. The results shown in the analysis are taken for the AES with 128-bit cryptography. From Table 3, Table 4 and Table 5, it is known that the designed AES-FJMKE consumes 1–67% of resources during the implementation. On the other hand, the operating frequency for the AES-FJMKE designed in the Virtex 5 is 751.247 MHz, which is higher than the other FPGA devices. The higher operating frequency is achieved by avoiding frequent computation of keys during the encryption/decryption processes. For verification purposes, the generation of multiple keys using a single input is illustrated in the simulation waveform of Figure 5. Here, the simulation waveform of key generation is taken for the Virtex 4 FPGA device. In Figure 5, the input is represented as W, and multiple subkeys are represented as k1–k10. From the analysis, we conclude that the FJMKE offers distinct subkeys for each input value. There is no similarity between the generated subkey values of FJMKE. Moreover, the overall simulation waveform for the AES-FJMKE architecture is shown in Figure 6. There, the plain text, secret key and cipher text are represented as plain, key, and cipher, respectively. The generated cipher text for the plain text with different secret keys is shown in Table 7. Table 7 shows that the AES-FJMKE architecture offers different cipher text for the same plain text according to the secret key. Hence, it is proved that the AES-FJMKE architecture offers higher robustness against the unauthorized users. Accordingly, the difficulty of accessing the data during the communication is difficult by the unauthorized users.

### 4.2. Comparative Evaluation

A comparison of the AES-FJMKE architecture is presented in this section. In this research, six different existing methods, namely AES-PNSG [41], AES-AT [42], typical AES [43], AES-BE [43], ISAES [44], AES-RS [45], and AES-MPPRM [46], are used to evaluate the AES-FJMKE architecture. Here, the evaluation is performed using six FPGA devices such as Virtex 4, Virtex 5, Virtex 6, Spartan 3, Spartan 6, and Kintex 7. The evaluation among the AES-FJMKE architecture and existing methods are provided as follows.

**Figure 7:**
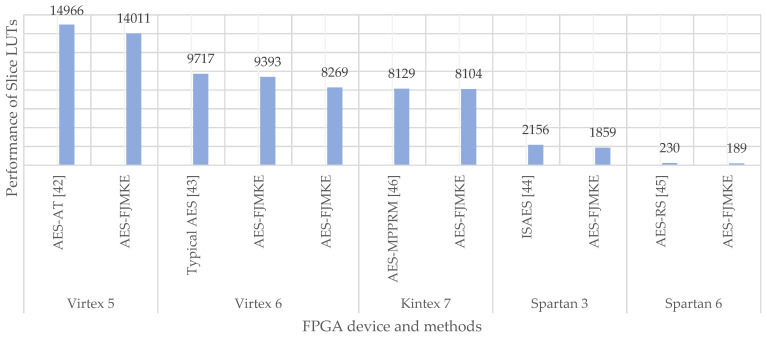
Graphical illustration of Slice LUTs.

Table 8, Table 9, Table 10, Table 11, Table 12 and Table 13 shows the evaluation of the AES-FJMKE with AES-PNSG [41], AES-AT [42], typical AES [43], AES-BE [43], ISAES [44], AES-RS [45], and AES-MPPRM [46], respectively. Table 8, Table 9, Table 10, Table 11, Table 12 and Table 13 compare the data of Virtex 4, Virtex 5, Virtex 6, Spartan 3, Spartan 6, and Kintex 7, respectively. Finally, the graphical illustration for the slice LUTs is shown in Figure 7. From the analysis, it is known that the AES-FJMKE architecture provides better performance in terms of hardware utilization, delay, and operating frequency. The slice registers of AES-FJMKE designed in the Virtex 6 is slightly high than the typical AES [43] and AES-BE [43]. From Table 10, it is noted that there are 2700 slice registers used in the AES-FJMKE architecture, which is higher than the number of slice registers of typical AES [23], i.e., 2688. However, the delay achieved by the AES-FJMKE architecture is 3.133 ns, which is less when compared to the typical AES [23], i.e., 3.205 ns. Hence, it is proved that the AES-FJMKE architecture achieves less delay than the typical AES architecture due to its concurrent generation of subkeys using FJMKE architecture. However, the increment in slice register is only at a negligible level that does not create any huge impact in terms of overall performances of AES-FJMKE. The reasons for the poor performance of existing architectures are specified as follows: the AES-PNSG [41] requires higher hardware resources, because of the non-pipelined stages-based AES implementation. Next, the AES-AT [42], AES-BE [43], and ISAES [44] have resulted in a high delay while encrypting and decrypting the plain texts. Further, the design of AES-MPPRM [46] requires a frequent computation of secret keys to accomplish encryption/decryption processes, which increases the hardware resources of the overall AES. However, a simultaneous generation of multiple keys using the AES-FJMKE architecture leads to minimize the delay as well as helps to reduce the combinational logics during the key generation. Therefore, FJMKE architecture is better than the existing AES architecture, because the generation of subkeys in the FJMKE does not consume much time as it is only depends on the main secret key. On the other hand, the subkey generation of conventional AES architectures mainly depends on the subkey from the previous step. So, the current subkey generation of conventional AES architectures has to wait until the completion of previous step subkey generation, which increases the propagation delay. Moreover, the key expansion using FJMKE is performed only one time during the encryption/decryption processes which further decreases the propagation delay. Therefore, the developed AES-FJMKE architecture achieved less delay and less hardware resources while maintaining the security of the plaintext. 

### 4.3. Case Study

**Figure 8:**
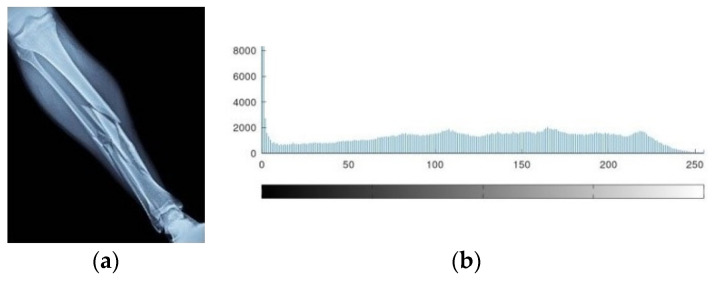
Input data, (**a**) Leg X-ray image, (**b**) Histogram.

**Figure 9:**
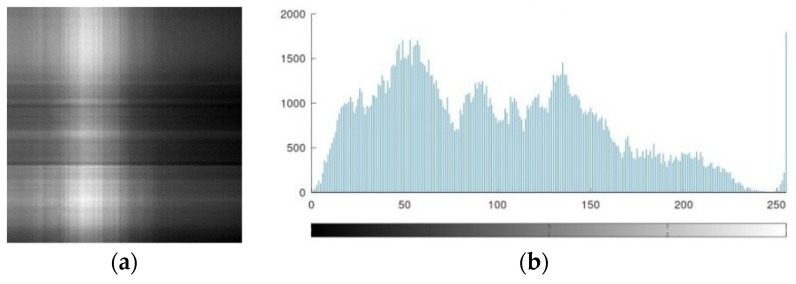
Output data, (**a**) encrypted image, (**b**) histogram.

The AES-FJMKE architecture is developed to achieve the secure broadcasting of the human leg X-ray image. The pixel value of the input image is obtained using the MATLAB 6.5 programming software whereas these pixels are used as input data for the AES-FJMKE architecture. Since the input pixels are in the range of 0 to 255, the input pixels are {6c, 9d,99,…, 4e}. Figure 8 shows the input X-ray image and its histogram representation. Next, the dec2bin command is used to convert the input pixel values into binary values which are encrypted using the AES-FJMKE architecture. From the AES-FJMKE, the output is acquired in a hexadecimal form that has an 8-bit size. Next, this ciphertext is securely broadcasted worldwide. Hence, the recovery of input data by an unauthorized person is difficult because of the effective encryption processed by the AES-FJMKE. The encrypted pixels are {fc, fc, fd, …, 2} and its corresponding encrypted and histogram image is shown in Figure 9. The ciphertext values are transformed into original pixel values in the receiving end by using the same AES-FJMKE architecture. Next, the decrypted pixels are given to MATLAB to develop the input image. From the encrypted and histogram images, it is concluded that the AES-FJMKE architecture offers a high level of security.

In FJMKE architecture, the generation of the subkeys mainly depends on the main key; therefore, the subkeys are simultaneously generated while accomplishing the encryption/decryption processes. Accordingly, the propagation delay is minimized for the overall architecture. However, the logical elements of the AES-FJMKE architecture is slightly higher than the typical AES architecture, whereas this slight increment in the resources does not create any huge impact in overall performances, because the designed key expansion using FJMKE is accomplished only one time during the encryption/decryption processes. Moreover, the designed FJMKE provides a unique subkeys for each input value; hence, there is similarity among the generated subkeys. Accordingly, the AES-FJMKE architecture provides high robustness against the unauthorized users.

## 5. Conclusions 

The purpose of this research is to propose a fast and efficient key-scheduling process for the AES algorithm. This AES-FJMKE architecture generates the required number of subkeys at a faster rate with minimal chip area sacrifice. This AES-FJMKE architecture ensures that the security of the messages processed does not affect the original plaintext. Therefore, the simultaneous multiple subkey generation using the AES-FJMKE architecture helps to decrease the delay. On the other hand, this multiple subkey generation is employed for decreasing the combinational logics by avoiding the frequent computation of subkeys. Hence, the AES-FJMKE architecture minimizes the delay and hardware utilization of the AES. Accordingly, the lesser delay in the computation process improves the operating frequency of the AES-FJMKE. The AES-FJMKE provides better performance than the AES-PNSG, AES-AT, typical AES, AES-BE, ISAES, AES-RS, and AES-MPPRM. The AES-FJMKE implemented using Spartan 6 FPGA used fewer slices (i.e., 76) than the AES-RS. However, without affecting the area’s consumption, the propagation delay of the key scheduling process can be further reduced. This can be accomplished by incorporating optimization techniques into other transformations, such as pipelined generation of subkeys and optimization of the temporary word generation process using the S-box implementation. The developed AES can be effectively encrypted and decrypt an entire nation’s sensitive passport information.

## Figures and Tables

**Table 1:** Types of AES algorithm.

AES Types	Key Sizes	Rounds (Nr)	No. of Key (Nr + 1)
AES-128	128	10	11
AES-192	192	12	13
AES-256	256	14	15

**Table 2:** R constant values for different rounds in AES–128.

Round	Rcon	Round	Rcon
1	(01000000)16	6	(20000000)16
2	(02000000)16	7	(40000000)16
3	(04000000)16	8	(80000000)16
4	(08000000)16	9	(1B000000)16
5	(10000000)16	10	(36000000)16

**Table 3:** Analysis of used resources for AES-FJMKE designed in Virtex devices.

Virtex FPGA Devices	FPGA Performances	Used Resources	Available Resources	Total Usage (%)
Virtex 4 FPGA	Number of slice registers	8452	10,944	77.22
	Flip Flops	8452	10,944	77.22
	Number of slice LUTs	7415	10,944	67.75
	Number of logical elements	7415	10,944	67.75
	Slices	3847	5472	70.3
	Bonded IOB	135	240	56.25
Virtex 5 FPGA	Number of slice registers	18,237	28,800	63.32
	Flip Flops	18,237	28,800	63.32
	Number of slice LUTs	14,011	28,800	48.64
	Number of logical elements	14,011	28,800	48.64
	Slices	4850	7200	67.36
	Bonded IOB	102	480	21.25
Virtex 6 FPGA	Number of slice registers	2700	93,120	2.89
	Flip Flops	2700	93,120	2.89
	Number of slice LUTs	8269	46,560	17.75
	Number of logical elements	8254	46,560	17.72
	Slices	966	11,640	8.29
	Bonded IOB	89	240	37.08

**Table 4:** Analysis of used resources for AES-FJMKE designed in Spartan devices.

Spartan FPGA Devices	FPGA Performances	Used Resources	Available Resources	Total Usage (%)
Spartan 3 FPGA	Number of slice registers	523	3840	13.61
	Flip Flops	541	3840	14.08
	Number of slice LUTs	1859	3840	48.41
	Number of logical elements	1859	3840	48.41
	Slices	972	1920	50.62
	Bonded IOB	58	141	41.13
Spartan 6 FPGA	Number of slice registers	78	18,224	1
	Flip Flops	81	18,224	1
	Number of slice LUTs	189	9112	2.07
	Number of logical elements	197	9112	2.16
	Slices	76	2278	3.33
	Bonded IOB	123	18,224	1

**Table 5:** Analysis of used resources for AES-FJMKE designed in Kintex 7 devices.

FPGA Performances	Used Resources	Available Resources	Total Usage (%)
Number of slice registers	7087	82,000	8.64
Flip Flops	7074	82,000	8.62
Number of slice LUTs	8104	41,000	19.76
Number of logical elements	8104	41,000	19.76
Slices	451	10,250	4.4
Bonded IOB	204	300	68

**Table 6:** Examination of delay and operating frequency for AES- FJMKE.

FPGA Devices	Delay (ns)	Operating Frequency (MHz)
Virtex 4	14.568	521.730
Virtex 5	2.402	751.247
Virtex 6	3.133	449.309
Spartan 3	3.229	101.491
Spartan 6	1.916	210.433
Kintex 7	2.540	97.308

**Table 7:** Results of cipher text for AES-FJMKE architecture.

Plain Text	Secret Key	Cipher Text
AD7532B3317176A831E2120013AA5481	2475A2B33475568831E2120013AA5481	515A192D6D2D880829A993A9D0D16F12
AD7532B3317176A831E2120013AA5481	6D353AA3B35952E831C611013AA5481	7C812B1650C6CDF37A793776497E58B2

**Table 8:** Evaluation of AES-FJMKE and AES-PNSG for Virtex 4.

Performances	AES-PNSG [41]	AES-FJMKE
Operating frequency (MHz)	214.48	521.730
Slices	20,818	2592

**Table 9:** Evaluation of AES-FJMKE and AES-AT for Virtex 5.

FPGA Performances	AES-AT [42]	AES-FJMKE
Operating frequency (MHz)	622.4	751.247
Slice LUTs	14,966	14,011
Slice registers	19,123	18,237
Slices	5974	4850

**Table 10:** Comparison of AES-FJMKE and AES-BE for Virtex 6.

Performances	Typical AES [43]	AES-BE [43]	AES-FJMKE
Operating frequency (MHz)	312.061	315.806	449.309
Delay (ns)	3.205	3.167	3.133
Slice LUTs	9717	9393	8269
Slice registers	2688	2688	2700

**Table 11:** Comparison of AES-FJMKE and ISAES for Spartan 3.

FPGA Performances	ISAES [44]	AES-FJMKE
Operating frequency (MHz)	67.75	101.491
Slices	1132	972
Slice LUTs	2156	1859
Flip Flops	680	541
IoB	389	58

**Table 12:** Comparison of AES-FJMKE and AES-RS for Spartan 6.

FPGA Performances	AES-RS [45]	AES-FJMKE
Operating frequency (MHz)	120	210.433
Slices	108	76
Slice LUTs	230	189
Flip Flops	92	81

**Table 13:** Comparison of AES-FJMKE and AES-MPPRM for Kintex 7.

Performances	AES-MPPRM [46]	AES-FJMKE
Operating frequency (MHz)	81.328	97.308
Delay (ns)	2.982	2.540
Slice registers	7120	7087
Flip Flops	7119	7074
Slice LUTs	8129	8104
Logical elements	8129	8104
Slices	467	451
Bonded IOB	211	204

## Data Availability

No new data were created or analysed in this study. Data sharing is not applicable to this article.
